# The prognostic significance of different proportion of signet-ring cells of colorectal carcinoma

**DOI:** 10.17305/bjbms.2021.5856

**Published:** 2021-05-29

**Authors:** Wei Chen, Huajun Cai, Kui Chen, Xing Liu, Weizhong Jiang, Shoufeng Li, Yiyi Zhang, Zhifen Chen, Guoxian Guan

**Affiliations:** 1 Department of Gynecology, The Affiliated Hospital of Putian University, Putian, China; 2 Department of Colorectal Surgery, The First Affiliated Hospital of Fujian Medical University, Fuzhou, China; 3 Department of General Surgery, The First Hospital of Fuzhou City Affiliated Fujian Medical University, Fuzhou, China; 4 Department of Colorectal Surgery, Fujian Medical University Union Hospital, Fuzhou, China

**Keywords:** Rectal cancer, signet-ring cell carcinoma, prognosis, nomogram

## Abstract

While the prognosis of patients with partial signet-ring cell carcinoma (PSRCC) has been rarely reported, colorectal signet-ring cell carcinoma (SRCC) has been associated with poor prognosis. The aim of this study was to analyze the prognosis of patients with different signet-ring cell (SRC) composition and establish a prediction model. A total of 91 patients with SRC component were included in the study. These patients were divided into two groups: SRCC group (SRC composition >50%; n = 41) and PSRCC group (SRC composition ≤50%; n = 50). COX regression model was used to identify independent prognostic factors for overall survival (OS). A predictive nomogram was established and compared with the 7^th^ American Joint Committee on Cancer (AJCC) staging system. After a median follow-up of 16 months, no significant difference in OS was observed in either group. Pre-operative carcinoembryonic antigen level, pN stage, M stage, pre-operative ileus, and adjuvant chemotherapy were independent prognostic risk factors for OS (*p* < 0.05). A nomogram for predicting the OS of colorectal SRCC was established with a C-index of 0.800, and it showed better performance than that of the 7^th^ AJCC staging system (*p* < 0.001). In summary, the ratio of SRC component was not an independent prognostic factor of the OS. Those patients with <50% of SRC component should be given the same clinical attention. A predictive nomogram for survival based on five independent prognostic factors was developed and showed better performance than the 7^th^ AJCC staging system. This resulted to be helpful for individualized prognosis prediction and risk assessment.

## INTRODUCTION

Signet-ring cell carcinoma (SRCC) is a rare histological subtype of colorectal cancer (CRC), which was first reported by Laufman and Saphir in 1951 [1]. According to the World Health Organization (WHO) criterion, SRCC is composed of more than 50% of signet-ring cells (SRCs) in which the nucleus is squeezed to the side by the prominent intracytoplasmic mucin [2]. The overall incidence of SRCC in colorectum is rare, ranging from 0.1% to 5% in Western countries [3-6] and 1.2-4.6% in China [7]. When compared with classical adenocarcinoma, SRCC is more likely to be associated with younger age at presentation, more advanced stage of diagnosis, and worse prognosis. Due to its rare occurrence, literature on colorectal SRCC is limited. However, distinct differences in clinicopathological manifestations make SRCC a unique entity that requires special attention.

Eventhough, the WHO defines SRCC as SRC that exceeds 50% of tumor tissue composition, in the daily clinical practice, we also encounter patients with tumors exhibiting a component of SRCs with less than 50% of the tumor mass. However, the clinical characteristics and prognosis of these patients are less well characterized, mainly due to the overall low incidence of colorectal SRCC. Whether the ratio of SRCs component plays a role in tumor biology and prognosis in SRCC still remains uncertain. The high-risk patients could be candidates for more individualized follow-up and intense adjuvant therapy. In addition, no individualized prognostic prediction model has yet been established for this subset of patients.

Therefore, the present study was aimed to compare the clinicopathological characteristics and prognosis of CRC patients with different SRC components, and to identify independent prognostic factors. Moreover, an individualized predicting model for the prognosis of CRC patients with different SRC component was established to help better prognostication and clinical decision-making.

## MATERIALS AND METHODS

### Patients

Clinicopathological data of patients with colorectal SRCC at Fujian Medical University Union Hospital from January 2010 to September 2018 were analyzed retrospectively. The inclusion criteria were as follows: (1) Pathologically proven SRCC; (2) surgical treatment, including radical and palliative surgery; and (3) complete clinical and pathological data. Exclusion criteria included (1) familial polyposis, ulcerative colitis, or Lynch syndrome; (2) multiple primary CRC or combined with other tumors; and (3) presence of other pathological components (e.g. neuroendocrine). Finally, a total of 91 patients were enrolled in our analysis.

### Definitions

**FIGURE 1:**
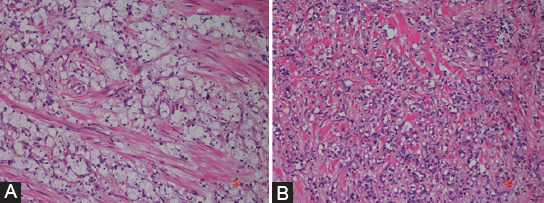
(A) SRCC (hematoxylin and eosin; ×200). (B) PSRCC. Signet-ring cells accounted for ≤50% of tumor composition (hematoxylin and eosin; ×200). SRCC: Signet-ring cell carcinoma; PSRCC: Partial signet-ring cell carcinoma.

**FIGURE 2:**
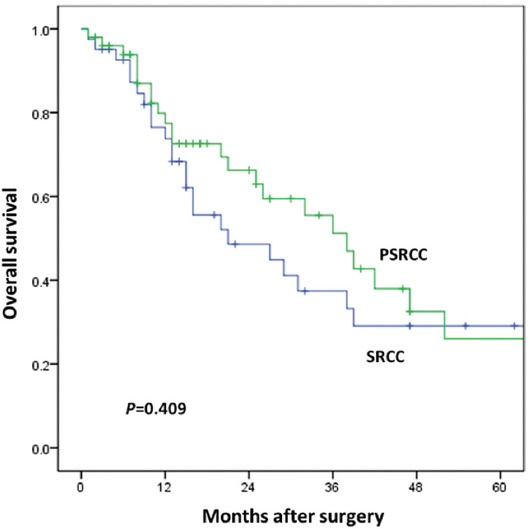
Overall survival between SRCC and PSRCC group. SRCC: Signet-ring cell carcinoma; PSRCC: Partial signet-ring cell carcinoma.

Patients were divided into two groups according to the percentages of SRCC and mucin: (1) SRCC group (SRC component >50%, Figure 1A) and (2) PSRCC group (SRC component ≤50%, Figure 1B). The pathological type and signet-ring cell amount and percentage were assessed by two experienced pathologists, independently. Pathological staging of the tumor was based on the 7^th^ edition of the American Joint Committee on Cancer (AJCC) tumor staging system. Tumor location was divided into proximal colon, distal colon, and rectum. Proximal colon was defined as ileocecum, cecum, ascending colon, and transverse colon up to the hepatic flexure. Distal colon was defined as splenic flexure, descending colon, sigmoid, and rectosigmoid colon. Palliative surgery included microscopical tumor remnants (R1) and macroscopical tumor remnants (R2) resection, bypass surgery, or ileostomy.

### Follow-up

All patients were followed up by an outpatient visit, telephone interview, WeChat, or email. The follow-up interval was once every 3 months in the first 2 years, once every 6 months in the following 3 years, and once a year thereafter. At each visit, all patients received imaging and laboratory examination, including tumor markers, such as carcinoembryonic antigen (CEA) and carbohydrate antigen 199 (CA199). In addition, chest radiographs, computed tomography or magnetic resonance imaging examinations, and endoscopy were performed at least once a year. Further assessment, such as positron emission tomography, was performed when required. The last follow-up date was September 30, 2018. Overall survival (OS) was measured as the time from the date of operation to the date of death or last contact.

### Statistical analysis

Statistical analysis was performed using SPSS 22. 0 (SPSS INC., Chicago). Categorical variables were compared by Chi-square or Fisher’s exact test. Continuous variables were compared by independent samples *t*-test. The survival rate was evaluated by the Kaplan–Meier method and compared by the log-rank test. Univariate and multivariate analysis of the independent prognostic factors of CRC with SRCC was performed using COX risk regression. Based on the independent prognostic factors in the COX regression model, a predicting nomogram was formulated using R 3.5.1 (www.r-project.org) with the survival and rms package. The discrimination of the nomogram was evaluated by calculating the Harrell’s concordance index (C-index). The higher C-index the better performance of the prediction model. The nomogram was internally validated by 1000 resampling bootstrap. The calibration plot was performed by comparing the predicted survival probability and the actual observation. According to the nomogram, the total score for each patient was calculated, and the first 75% of the score was identified as cutoff value using the quartile spacing method. Risk classifications were illustrated with the Kaplan–Meier curve after the patients were divided into different prognostic groups according to percentile scores. The former 75% of the patients were classified as the low-risk group, the remaining patients as the high-risk group. To evaluate the predictive performance of the 7^th^ AJCC staging system and our prediction model, the receiver operating characteristic (ROC) curve was drawn and the area under the ROC curve (AUC) was calculated. *p* < 0.05 was considered statistically significant.

## RESULTS

### Patient and pathological characteristics

A total of 91 patients with colorectal SRCC were enrolled in our analysis, including 41 patients with SRCC and 50 patients with PSRCC. There were 53 (58.2%) males and 38 (41.8%) females with a median age of 52.3 years. The clinicopathological characteristics of the two groups are shown in Table 1. No significant difference was observed between the two groups in terms of age, sex, tumor location, and pathological stage (all *p* > 0.05, Table 1). Among them, 54/91 (59.3%) patients had tumors located in the colon, while 37/91 (40.7%) had tumors located in the rectum. The distribution of sites for primary tumors was similar in SRCC and PSRCC groups (*p* = 0.165). Patients in both groups presented with larger tumors (6.1 ± 2.1 vs. 6.1 ± 2.2, *p* = 0.455) and mostly ulcerative lesion (59% vs. 58%, *p* = 0.495). Patients in the PSRCC group were more likely to present with higher pre-operative CEA and CA199 levels, when compared with SRCC patients (*p* = 0.029 and *p* = 0.017, respectively). Interestingly, we observed a higher probability of pre-operative ileus in patients in the SRCC group (41.5% vs. 14%, *p*<0.003). A higher proportion of patients with pT3-4 (86/91, 94.5%), pN2 (63/91, 69.2%), and III-IV stage (80/91, 87.9%) disease was noted in our study cohort. There was no significant difference in pT stage, pN stage, and pathological staging between the two groups (all *p* > 0.05).

**TABLE 1:**
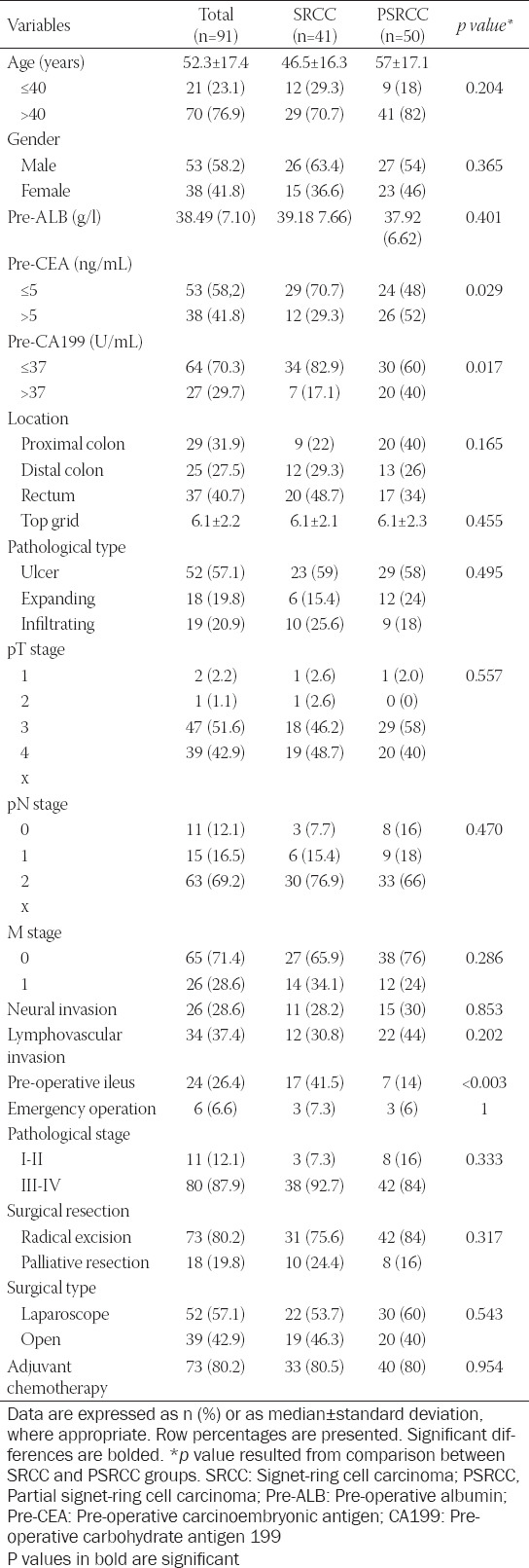
Clinicopathological characteristics of SRCC colorectal cancer patients

### Survival outcomes

The median follow-up time was 16 months (range 1-107). In our study cohort, 52/91 (57.1%) patients experienced tumor recurrence, including peritoneal metastases (33/91, 36.3%). The 1-, 2-, and 3-year OS rates in SRCC and PSRCC group were 73.8% versus 77.4%, 48.6% versus 66.3%, and 37.4% versus 51.2%, respectively. However, this difference was not statistically significant, as demonstrated in the Kaplan–Meier survival curve (*p* = 0.409, Figure 2).

### Prognostic factors of OS

Univariate analysis showed that the prognostic risk factors of OS included pre-operative CEA level (hazard ratio [HR] = 2.278, 95% confidence interval [CI] 1.269-4.090, *p* = 0.006), pre-operative albumin level (HR = 0.929, 95% CI 0.891-0.969, *p* = 0.001), tumor size (HR = 1.273, 95% CI 1.072-1.427,*p* = 0.004), pT stage (HR = 2.170, 95% CI 1.236-3.811, *p* = 0.007), pN stage (HR = 2.204, 95% CI 1.185-4.099, *p* = 0.013), pM stage (HR = 2.475, 95% CI 1.376-4.453, *p* = 0.002), pre-operative ileus (HR = 4.394, 95% CI 2.334-8.276, *p* = 0.000), emergency surgery (HR = 7.564, 95% CI 3.016-18.975, *p* < 0.001), surgical resection (HR = 2.308, 95% CI 1.223-4.356, *P* = 0.010), surgical type (HR = 2.428, 95% CI 1.345-4.382, *p* = 0.003), and adjuvant chemotherapy (HR = 0.496, 95% CI 0.251-0.981, *p* = 0.004), as shown in Table 2.

**TABLE 2:**
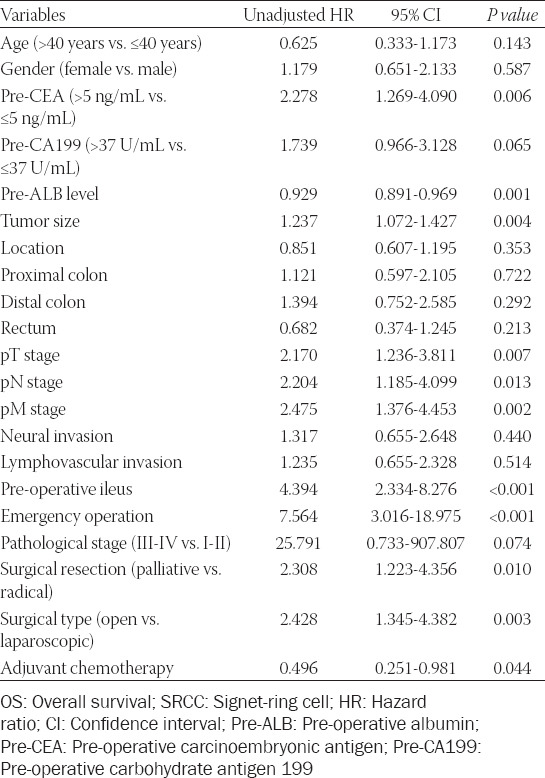
Univariate analysis of risk factors of OS in SRCC colorectal cancer

After adjusting for confounding factors, COX regression analysis demonstrated that the independent risk factors for overall survival were pre-operative CEA level (HR = 3.286, 95% CI 1.584-6.819, *p* = 0.001), pN stage (HR = 2.618, 95% CI 1.282-5.345, *p* = 0.008), M stage (HR = 2.804, 95% CI 1.165-6.747, *p* = 0.021), pre-operative ileus (HR = 5.457, 95% CI 2.142-13.900, *p* < 0.001), and adjuvant chemotherapy (HR = 0.153, 95% CI 0.064-0.362, *p* < 0.001), as demonstrated in Table 3.

**TABLE 3:**
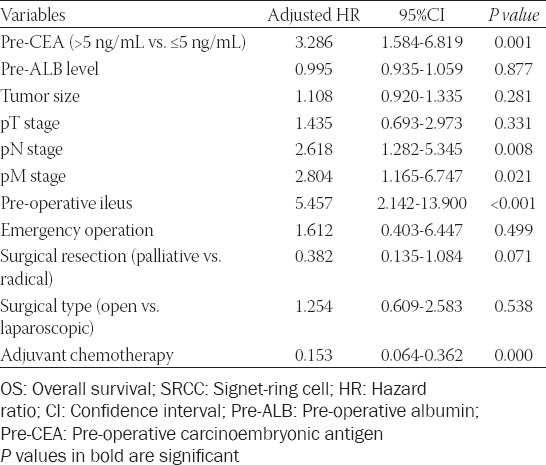
Multivariate analysis of risk factors of OS in SRCC colorectal cancer

### Establishment and evaluation of a nomogram prediction model

**FIGURE 3:**
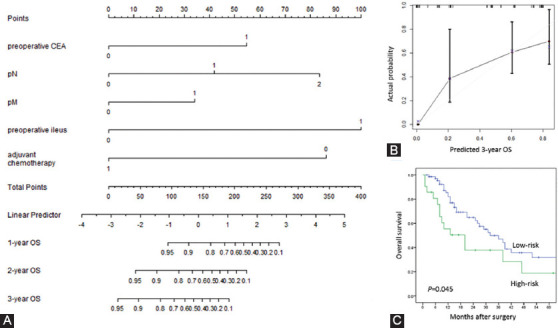
(A) Nomogram predicting the probability of 1-, 2-, and 3-year overall survival in SRCC colorectal cancer patients. (B) Calibration curves for 3- and 5-year disease-free survival in the internal validation cohort. (C) Overall survival between low-risk and high-risk groups. SRCC: Signet-ring cell carcinoma.

Based on the significant independent factors in the COX regression model, a nomogram was established for predicting OS in CRC patients with SRC component, as shown in Figure 3A. To determine the probability of survival, total score was obtained by summing up points for each variable. Patients with a higher total score were more likely to obtain a lower probability of 1-, 2-, and 3-year OS. The performance of the nomogram for OS prediction was bootstrapped and validated internally with a C-index of 0.800. The calibration plot for the probability of 3-year OS presented good agreement between predicted probability and actual observation (Figure 3B).

Each patient was scored and ranked according to percentiles, and afterward, risk classifications and stratifications were implemented to illustrate the ability of the nomogram to make risk assessments. The first 75% was identified as the cutoff value using the quartile spacing method, and the remainder was defined as high-risk and low-risk group. As shown in Figure 3C, patents in the low-risk group were likely to experience a better probability of OS (*p* = 0.045), in accordance with results from our nomogram.

**FIGURE 4:**
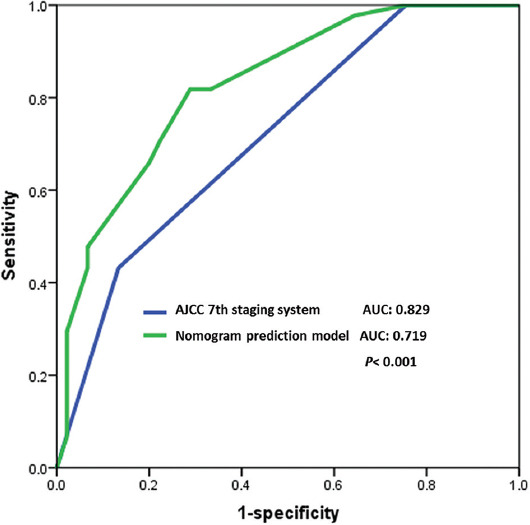
ROC curve for the nomogram prediction model and the AJCC 7^th^ staging system. ROC: Receiver operating characteristic; AJCC: American Joint Committee on Cancer.

In addition, the predictive value of our nomogram was compared with that of the 7^th^ AJCC staging system by drawing a ROC curve and calculating AUC. The AUC of the nomogram was 0.829, significantly higher than 0.719 of the 7^th^ AJCC staging system (*p* < 0.001). As illustrated in Figure 4, the nomogram showed superior OS predictive ability than the 7^th^ AJCC staging system.

## DISCUSSION

To date, only a few studies have focused on the clinical and prognostic value of the ratio of SRC components in colorectal SRCC. In the present study, we demonstrated that colorectal SRCC with different SRC component has similar clinicopathological characteristics and comparable oncological outcome. After adjusting for potential confounding factors, the ratio of SRC component was not an independent prognostic factor of the OS. In addition, we developed a predictive nomogram for survival, which showed better performance than the 7^th^ AJCC staging system.

In this study, the median age of CRC patients with different SRC components was 51, 46.5 in SRCC group and 57 in PSRCC group. It has been reported that colorectal SRCC is more likely to present at a younger age, which makes our results consistent with the previous studies [8-10]. Similarly, colorectal SRCC was more likely to locate in the proximal colon and rectum, which was similar to the previous studies [7,10-13]. For PSRCC patients, most clinicopathological characteristics were similar to those for SRCC patients. Interestingly, we noted that patients in the SRCC group were more likely to experience pre-operative ileus, which turned out to be an independent prognostic factor. Unfortunately, no available studies exist regarding whether the ratio of SRC component is associated with pre-operative ileus. One possible explanation for absence of studies may be due to the relatively larger tumor size and diffusely infiltrated growth pattern of SRCC. In our cohort, the proportion of elevated pre-operative CEA and CA199 was not high, and this will probably underestimate pretreatment risk stratification of tumors. Extensive intraperitoneal dissemination was often encountered during the operation which increased rates of conversion from laparoscopic to open surgery. Our study suggested that the behavior pattern of PSRCC tumors might be similar to that of SRCC tumors.

It has been well documented that colorectal SRCC is associated with poor prognosis, with the 5-year survival rate ranging 0-37% [14-18]. Fu et al. [19] have demonstrated that the 5-year survival rate of resectable metastatic colorectal SRCC was 9.66%, which is significantly lower than that of normal adenocarcinoma and mucinous adenocarcinoma. In addition, molecular biological characteristics of colorectal SRCC were reported to be similar to those of standard SRCC [20]. There are inconclusive results regarding the prognostic value of the SRC ratio in colorectal SRCC. Inamura et al. [10] found that <50% of the SRC component was associated with a poorer prognosis and independent of other clinicopathological and tumor molecular characteristics. Tan et al. [7] have shown that the prognosis of colorectal SRCC is poor regardless of the SRC component. Similarly, our results demonstrated that colorectal SRCC patients with different signet-ring cells components had similar OS. After adjustment for other confounding factors, the SRC ratio was not an independent predictor of the overall outcome.

Indeed, the molecular characterization may contribute to better understanding of SRCC. The previous studies demonstrate that colorectal SRCC exhibits features of aberrant DNA methylation [21]. Proximal colorectal SRCC is characterized by hypermethylated, with CpG island methylator phenotype, BRAF V600E mutation, and microsatellite instability [22]. On the other hand, distal colorectal SRCC is characterized by hypomethylated and upregulation of the epithelial-mesenchymal transition gene expression [22], all of which help to explain invasion and metastasis of tumor.

Similar to the previous studies, our study identified several independent prognostic factors of OS, including pre-operative CEA level, pN stage, M stage, pre-operative ileus, and adjuvant chemotherapy. Hugen et al. [11] have found that colorectal SRCC may benefit from adjuvant chemotherapy despite its dismal prognosis. In our study, the prognosis of patients with SRCC receiving adjuvant chemotherapy was better than those without adjuvant chemotherapy (*p* < 0.038). Univariate and multivariate analysis identified adjuvant chemotherapy as an independent risk factor for prognosis (*p* < 0.001). A high incidence of peritoneal dissemination has been noted in colorectal SRCC in clinical practice. At present, cytoreductive surgery with hyperthermic intraperitoneal chemotherapy (CRS-HIPEC) is generally recommended as a treatment option for peritoneal carcinomatosis from digestive system cancers. Simken et al. [23] showed that SRCC is an adverse prognostic factor for intraperitoneal infusion chemotherapy (HR = 3.73, 95% CI 1.88-7.41) and that patients with peritoneal metastasis from CRC can benefit from intraperitoneal infusion chemotherapy. Unfortunately, the therapeutic effect and complications of CRS-HIPEC in our cohort could not be evaluated due to the lack of data.

Nomograms, as a new type of prediction tool, are currently widely used in clinical prediction for CRC patients [24-26]. We developed a predictive nomogram for OS in CRC with different SRC components by incorporating the five independent risk factors in the COX regression model. The prediction model enables us to generate an individualized prediction by a combination of five easy to obtained risk factors of colorectal SRCC in clinical practice. Moreover, we further evaluated the ability of the nomogram to make a risk assessment. The results showed that patents in the low-risk group were likely to experience a better probability of OS (*p* = 0.045). Therefore, our nomogram is helpful for clinicians to identify high-risk patients who are candidates for more intense follow-up and adjuvant therapy.

At present, the AJCC cancer staging system is the gold standard for predicting the prognosis of CRC patients [27,28]. Herein, we evaluated whether the AJCC staging system is effective in predicting the prognosis of CRC patients with different SRC components, as compared to our predictive nomogram. The results showed that the accuracy and predictive ability of our predictive nomogram were significantly better than those of the 7^th^ AJCC staging system. Nevertheless, performance of our nomogram warrants further validation in larger population. There are several limitations in the present study. First, our study is a single-institutional retrospective study, which may be subject to selective bias. Second, due to the lack of adequate data, our study could not provide insight into the molecular and biological characteristics of CRC with different SRC components. Third, our study did not include clinical symptoms and physical status scores, and failed to evaluate the efficacy of different chemotherapy regimens. In addition, the prediction model is only validated internally due to relatively small sample size, and external validation in a large patient cohort is warranted. Despite these limitations, our study shed light into CRC with different SRC components in terms of clinicopathological characteristics and oncological outcome. To the best of our knowledge, for the 1^st^ time, an individualized prognostic model was established for CRC patients with different SRC components in our study, and its predictive ability was significantly higher than that of the traditional AJCC staging system.

## CONCLUSION

Our results demonstrated that colorectal SRCC has similar clinicopathological characteristics and comparable oncological outcome, despite the ratio of SRC components. After adjusting for potential confounding factors, ratio of SRC component was not an independent prognostic factor of the OS. Pre-operative CEA level, pN stage, M stage, pre-operative ileus, and adjuvant chemotherapy remained to be independent prognostic factors of OS. In addition, we developed a predictive nomogram for survival, which showed better performance than the 7^th^ AJCC staging system.

### Statement of ethics

This study was approved by the Medical Ethics Committee of our hospital.
